# Synthesis of π-Conjugated Polymers Containing Benzotriazole Units via Palladium-Catalyzed Direct C-H Cross-Coupling Polycondensation for OLEDs Applications

**DOI:** 10.3390/polym13020254

**Published:** 2021-01-14

**Authors:** Dong Han, Jingwen Li, Qiang Zhang, Zewang He, Zhiwei Wu, Jingting Chu, Yan Lu

**Affiliations:** 1 School of Materials Science & Engineering, Tianjin University of Technology, Tianjin 300384, China; hl912928@163.com (D.H.); yixialjw@126.com (J.L.); JEFFHZW1995@163.com (Z.H.); wuzhiwei2021@163.com (Z.W.); chu945699645@126.com (J.C.); 2 Tianjin Key Laboratory for Photoelectric Materials and Devices, Tianjin University of Technology, Tianjin 300384, China; 3 Key Laboratory of Display Materials & Photoelectric Devices, Ministry of Education, Tianjin University of Technology, Tianjin 300384, China

**Keywords:** C-H cross-coupling, D-π-A conjugated polymer, benzotriazole, OLEDs

## Abstract

Four D-π-A conjugated polymers, namely P1–P4, which contain benzotriazole building blocks in their backbone as acceptor, are synthesized via palladium-catalyzed direct C-H cross-coupling polycondensation of 5,6-difluorobenzotriazole with different thiophene derivatives, including 3-octylthiophene, 2,2’-bithiophene, thieno[3,4-b][1,4]dioxine, and 4,4-dioctyl-4H-silolo-[3,2-b:4,5-b’]dithiophene as donor units, respectively. Taking the polymer P1 as an example, the chemical structure of the polymer is demonstrated by ^1^H and ^19^F NMR spectra. The optical, electrochemical, and thermal properties of these polymers are assessed by UV–vis absorption and fluorescence spectroscopy, cyclic voltammetry (CV), and thermal gravimetric analysis (TGA), respectively. DFT simulations of all polymers are also performed to understand their physicochemical properties. Furthermore, P1 and P2, which have relatively higher molecular weights and better fluorescent quantum efficiency than those of P3 and P4, are utilized as lighting emitters for organic light-emitting diodes (OLEDs), affording promising green and red luminescence with 0.07% and 0.14% of maximum external quantum efficiency, respectively, based on a device with an architecture of ITO/PEDOT:PSS/PTAA/the polymer emitting layer/TPBi/LiF/Al.

## 1. Introduction

Organic light-emitting diodes (OLEDs) have received much attention both in academia and industry owing to their wide applications in fullcolor displays and solid-state lighting. π-Conjugated polymers have many unique photophysical properties that are inherited from the delocalized electronic structures of their backbones and, thus, they exhibit promising advantages, such as solution-processing, light-weight, low-cost, and facile preparation of flexible devices in various functional applications, such as OLEDs, solar cells, field-effect transistors (FETs), photodetectors, etc. [1,2,3,4,5,6].

A variety of π-conjugated polymers have been synthesized based on reliable coupling methods including Suzuki–Miyaura [7], Stille [8], and Kumada–Corriu couplings [9], etc. For example, Todd and coworkers used brominated 5,6-difluorobenzotriazole and 4-hexylthiophene as starting materials and iPrMgCl·LiCl as Grignard reagents to synthesize the alternating polymer poly(5,6-difluorobenzotriazole-alt-4-hexylthiophene) via Kumada catalyst-transfer polycondensation [10]. In 2014, several benzotriazole-containing π-conjugated polymers were also achieved through Stille coupling reaction of 4,7-dibromo-5,6-difluorobenzotriazole with 2,5-bis(trimethylstannyl)thiophene derivatives for polymer field-effect transistors (FETs) applications [11]. These synthetic methods above for benzotriazole-based π-conjugated polymers generally require halogenations and/or organometallic pre-functionalization steps of the corresponding monomers prior to polymerization. It is often difficult to synthesize and purify the metalated monomers due to their instability. In addition, in some cases, these polymerization processes can produce a toxic byproduct, for example, trialkyltin bromide. To circumvent these problems, direct arylation polymerization (DArP) has been developed, exhibiting several advantages, including fewer synthetic steps, easier purification of monomers, and reduced environmental pollution. For instance, Luscombe et al. successfully synthesized the alternating copolymer of 5,6-difluorobenzotriazole and 2,5-dithienylsilole with Mn = 10,000 via DArP [12]. Most recently, transition-metal catalyzed direct C-H coupling strategy was proposed and has been applied for preparing various π-conjugated polymers, including our work [13,14,15,16,17]. This synthetic protocol fully avoids the steps of pre-functionalized starting monomers, and allows straightforward access to π-conjugated polymers [13,14,15,16,17,18,19,20,21,22,23,24,25,26,27,28]. However, this method is mainly limited to the homo-coupling of some aromatic monomers such as thienopyrroledione (TPD) [13], bithiazole [14], benzodiimidazole [25,26,27], and ester-functionalized thiophene [16,17,18,19] at present. Obviously, it is more challenging to achieve the highly selective cross-coupling of two different aromatic monomers as there are many possible multiple coupling patterns.

In this work, a series of D-π-A type conjugated polymers containing benzotriazole as acceptor units were successfully synthesized via Pd-catalyzed direct C-H cross-coupling polycondensation reaction of 5,6-difluorobenzotriazole with thiophene derivatives. Furthermore, the as-prepared polymers’ physicochemical properties and light-emitting performance were also assessed.

## 2. Experimental

### 2.1. Materials

5,6-Difluoro-2-(2-hexyldecyl)-2*H*-benzo[*d*][1,2,3]triazole, 3-octylthiophene, 2,2’-bithiophene, thieno[3,4-*b*][1,4]dioxine, 4,4-dioctyl-4*H*-silolo[3,2-b:4,5-b]dithiophene, palladium acetate (Pd(OAc)_2_), pivalic acid (PivOH), potassium carbonate (K_2_CO_3_), and silver carbonate (Ag_2_CO_3_) were purchased from Zhengzhou HQ Material Co., Ltd. (Zhengzhou, China) for direct use. *N*,*N*-Dimethylacetamide (DMAc) and xylene were obtained from J&K Chemical (Beijing, China) and used without further purification. Poly(sodium-p-styrenesulfonate) (PSS), poly(2,3-dihydrothieno-1,4-dioxin) (PEDOT), poly[bis(4-phenyl)(2,4,6-trimethylphenyl)amine] (PTAA), and 1,3,5-tris(1-phenyl-1H-benzimidazol-2-yl)benzene (TPBi) were provided by Xi’an Polymer Light Technology Co. (Xi’an, China).

### 2.2. Instruments

^1^H and ^13^C NMR spectra of polymers were measured on a Bruker AV400 at 25 °C utilizing the residual solvent peak as reference. Molecular weights of polymers were determined by gel permeation chromatography (GPC) on Waters 1525 equipped with Waters Styragel HT gel columns using tetrahydrofuran (THF) as eluent at 35 °C and monodisperse polystyrene as standard. Thermogravimetric analyses (TGA) were carried out on a Frontier Mid-IR FTIR/STA6000-TL9000-Clarus SQ8 instrument with a 10 °C·min^−1^ heating rate under a purified nitrogen gas flow. Optical properties of polymers including UV–vis absorption and photoluminescence (PL) spectra were obtained on a Shimadzu UV-2550 and Hitachi F-4600, respectively. Solid-state samples for the test of photophysical property were prepared by spin-casting of polymers chloroform solutions on quartz plates. Optical bandgaps were calculated based on the onset of the absorption spectra of the thin film. Cyclic voltammetry (CV) measurements are carried out on a LK98B II electrochemical analyzer at room temperature with the scan rate was 100 mV s^−1^. In this experiment, a conventional three-electrode configuration was adopted, i.e., a glassy carbon electrode as the working electrode, a saturated calomel electrode (SCE) as the reference electrode, and a Pt wire as the counter electrode. Acetonitrile, freshly prepared (CH_3_CN) by distilling from calcium hydride (CaH) under dry N_2_, was used as solvent. Tetrabutylammonium phosphorus hexafluoride (Bu_4_NPF_6_) with a concentration of 0.1 M in CH_3_CN was applied as the support electrolyte.

### 2.3. Synthesis of Polymer

The polymers were prepared according to a similar procedure as reported in our recent work [27]. In detail, 5,6-difluoro-2-(2-hexyldecyl)-2*H*-benzo[*d*][1,2,3]triazole (**1**) (113.7 mg, 0.3 mmol), 3-octylthiophene (58.8 mg, 0.3 mmol) were dissolved in 3 mL of DMAc/xylene (*v/v*, 10/1). Silver carbonate (248.2 mg, 0.9 mmol) and potassium carbonate (124.4 mg, 0.9 mmol), pivalic acid (61.3 mg, 0.6 mmol), Pd(OAc)_2_ (6.7 mg, 10 mol%) were added into the above solution. The mixture was stirred for 48 h at 110 °C under N_2_. After the mixture was cooled to room temperature, it was poured into cold methanol (100 mL). The precipitate was collected and subsequently subjected to successive Soxhlet extractions with anhydrous methanol and hexanes for 24 h to remove possible catalytic residues and oligomers. The residue was then extracted with chloroform for another 12 h. The CHCl_3_ solution was concentrated to about 2 mL using a rotary evaporator and was then poured into methanol (60 mL). The precipitate was collected and dried under vacuum at 30 °C for 12 h to provide **P1** as a black solid (159 mg, 93 % yield). *M_n_* = 22.3 kDa, PDI = 1.82 (GPC). ^1^H NMR (400 MHz, CDCl_3_) *δ* 8.38 (m, *J* = 10.5 Hz, 1H), 4.86-4.37 (m, 2H), 2.70 (s, 2H), 2.28 (s, 1H), 1.68 (s, 2H), 1.45-1.04 (m, 34H), 0.84 (s, 9H).

**P2**, **P3**, and **P4** were synthesized based on a similar procedure as that for **P1**, except for the use of different monomers. **P2**: 131 mg, 82% yield, *M_n_* = 28.1 kDa, PDI = 1.22 (GPC). ^1^H NMR (400 MHz, CDCl_3_) *δ* 8.21 (m, 1H), 6.98 (br, 3H), 4.71 (d, 2H), 2.25 (s, 1H), 1.32 (d, 32H), 0.86 (s, 8H). **P3**: 15.6 mg, 10% yield, *M_n_* = 15.0 kDa, PDI = 1.59 (GPC). ^1^H NMR (400 MHz, CDCl_3_) *δ* 4.75-3.96 (m, 6H), 2.25 (s, 1H), 1.41 (d, 24H), 0.87 (d, *J* = 7.2 Hz, 6H). **P4**: 47.8 mg, 20% yield, *M_n_* = 3.5 kDa, PDI = 1.34 (GPC). ^1^H NMR (400 MHz, CDCl_3_) *δ* 6.98 (br, *J* = 15.4 Hz, 2H), 4.60 (d, 2H), 2.25 (s, 1H), 1.73-0.97 (m, 52H), 0.86 (s, 12H).

### 2.4. Device Fabrication and Characterization

The OLED devices with **P1** and **P2** as a light-emitting layer were fabricated with the following configuration: ITO/PSS:PEDOT/PTAA/polymer light-emitting layer/TPBi/LiF/Al.

In this experiment, indium tin oxide (ITO) glass with a conductivity of 10 Ω/square was first pre-cleaned with acetone and ethanol under ultrasound and was subsequently treated in an ultraviolet–ozone chamber. A thin 40 nm-thick PSS-PEDOT layer was spin-coated onto the treated ITO glass which was placed on a hot plate at 3000 rpm for 10 min at 110 °C. The coated ITO glass was then transferred to a glovebox filled with N_2_ and re-dried for 10 min at 110 °C. The solution of PTAA (5 mg) in 1 mL of DMF was then spin-coated onto the top of PSS:PEDOT layer on ITO to form an electron blocking layer with a thickness of 30 nm. After the above substrate was baked for 20 min at 100 °C, the toluene solution of **P1** or **P2** (10 mg/mL) was spin-coated onto PTAA layer to form a uniform 40 nm-thick light-emitting layer, which was followed by an annealing treatment for 10 min at 90 °C. Finally, using conventional thermal evaporation under vacuum, TPBi, LiF, and Al were deposited onto the surface of the active layer in sequence with the thicknesses of 30, 1, and 120 nm, respectively. The devices provided an active area of 5 × 3 mm^2^. Based on the above devices, the electroluminescence (EL) spectra, current density–voltage (*J*–*V*) and luminance–voltage (*L*–*V*) were obtained on a Keithley 2400 Source Meter system. The luminance of OLEDs was measured with a PhotoResearch SpectraScan PR-650 Colorimeter.

## 3. Results and Discussion

### 3.1. Synthesis and Characterization of Polymers

**Scheme 1:**
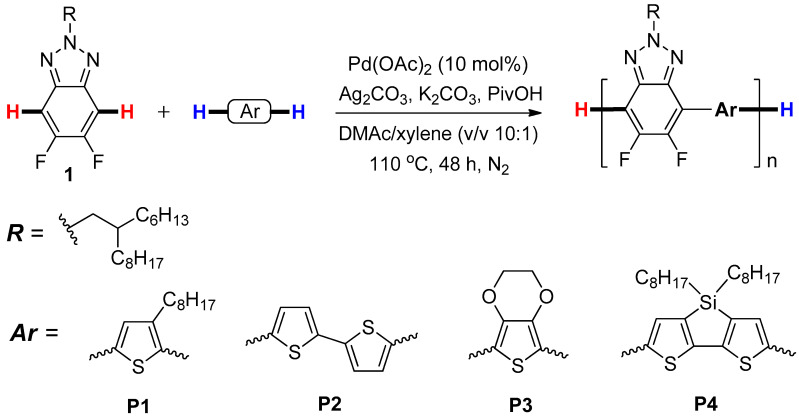
Synthetic routes of the polymers **P1**–**P4**.

As depicted in Scheme 1, **P1**–**P4** were prepared by Pd-catalyzed direct C-H cross-coupling reaction of 5,6-difluoro-2-(2-hexyldecyl)-2H-benzotriazole (**1**) with four different thiophene derivatives, including 3-octylthiophene, 2,2’-bithiophene, thieno[3,4-*b*][1,4]dioxine, and 4,4-dioctyl-4*H*-silolo-[3,2-b:4,5-b’]dithiophene under the optimized conditions as reported in our recent work [29], namely, Pd(OAc)_2_ (10 mol%) as catalyst, Ag_2_CO_3_ (3.0 equiv) as oxidant, K_2_CO_3_ (3.0 equiv) as base, PivOH (2.0 equiv) as additive, and DMAc/xylene (*v/v*, 10/1) as solvents at 110 °C for 48 h. A branched side chain (2-hexyldecyl) was attached to benzotriazole units, aiming to improve the solubility of compound **1** and the resultant copolymers in the common organic solvents, such as DMAc, DMF, CHCl_3_, etc. [11]. The results of the polycondensation reactions and the thermophysical properties of **P1**–**P4** were shown in Table 1.

The polycondensation of 5,6-difluoro-2-(2-hexyldecyl)-2H-benzotriazole (**1**) with 3-octylthiophene provides the polymer **P1** with an average molecular weight of *M*_n_ = 22,300 in an excellent yield (93%). In the case of 2,2’-bithiophene as donor units, **P2** with the highest molecular weight of *M*_n_ = 28,100 was obtained in a good yield (82%). By contrast, the polymerization reaction of **1** with thieno[3,4-*b*][1,4]dioxine mainly yielded an insoluble solid in common organic solvents, and only a small number of products can be isolated by Soxhlet extractions with CHCl_3_ to provide a total yield of about 10%. This result may be due to the cross-linking reaction in multiple C-H systems [30,31]. Dithienosilole cores are common building blocks of various active materials used in photovoltaics, FETs, and OLEDs, etc. [32,33,34,35,36] because of their strong donating electron ability, photostability, and chemical stability. Under the same reaction conditions as that for **P1**–**P3**, the polycondensation of **1** with 4,4-dioctyl-4*H*-silolo-[3,2-b:4,5-b’]dithiophene yields a soluble polymer **P4** in CHCl_3_ with low yield (about 20%) (Table 1, entry 4), which is similar to the case of **P3**. Most of the as-prepared polymers exhibit good thermal stability with the degradation temperatures at 5% weight loss are 385, 317, and 392 °C for **P1**, **P2**, and **P4**, respectively (Table 1), which is suitable for the applications in the semiconductor devices.

### 3.2. Structural Characterization

**Figure 1:**
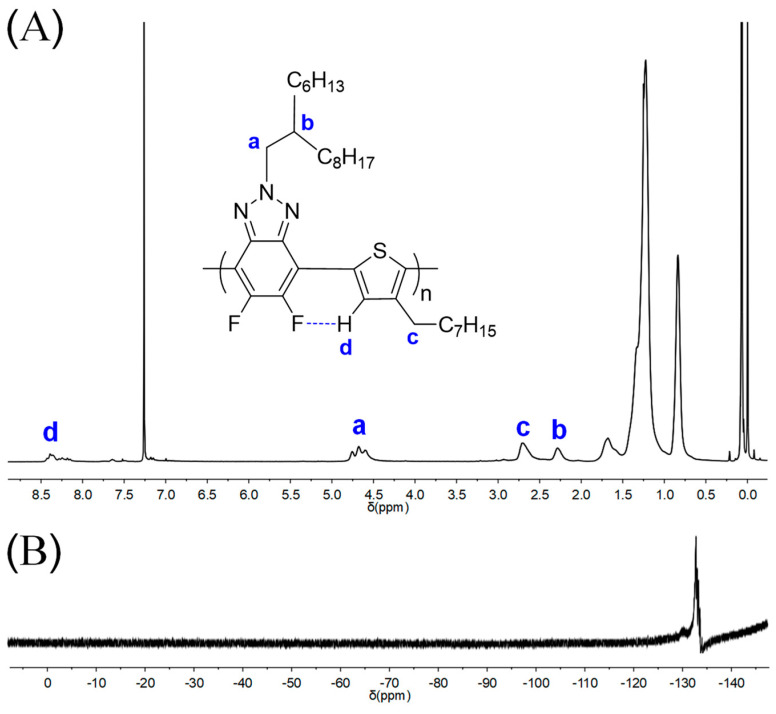
^1^H NMR (**A**) and ^19^F NMR (**B**) of **P1** in CDCl_3_.

The chemical structure of polymers was determined by ^1^H and ^19^F NMR spectra (Figure 1 and Figures S1–S8). Taking **P1** as an example, all characteristic proton peaks corresponding to the benzotriazole and thiophene units can be observed as shown in Figure 1A, which is similar to the analogous polymers obtained via the Kumada method except for different alkyl side chains as reported by Bielawski and co-workers [10]. As seen in Figure 1A, the multiple peaks at 4.60–4.76 ppm (a in Figure 1A) and the broad peak at 2.28 ppm (b in Figure 1A) were assigned to the -C*H*_2_ groups close to the triazole ring and -C*H* signal of the branched groups, respectively. The peak at 2.70 nm (c in Figure 1A) corresponds to -C*H*_2_ groups of thiophene side chains. It was noted that the signal of aromatic hydrogen of thiophene units (d in Figure 1A) significantly shifted to low field compared with the corresponding thiophene monomer, which was possibly caused by F⋯H noncovalent interaction between the fluorine atoms on benzotriazole unit and the hydrogen atoms on thiophene units as demonstrated by our recent work [29] and the literature [11].

Besides, ^19^F NMR was also applied to confirm the polymers’ chemical structure since it has many merits, including high sensitivity, little interference, a large range of chemical shifts, and similar structures that are not easy to overlap, etc. As depicted in Figure 1B, the ^19^F NMR of **P1** displayed an obvious main peak at −133.0 ppm that corresponds to the fluorine atoms on the polymeric chains. The remaining weak signals could be assigned to the end groups, where the fluorine atoms are not chemically equivalent to those along the backbone [37]. All the NMR results of **P1** suggest an alternating structure along the polymer main chain.

### 3.3. DFT Simulation

**Figure 2:**
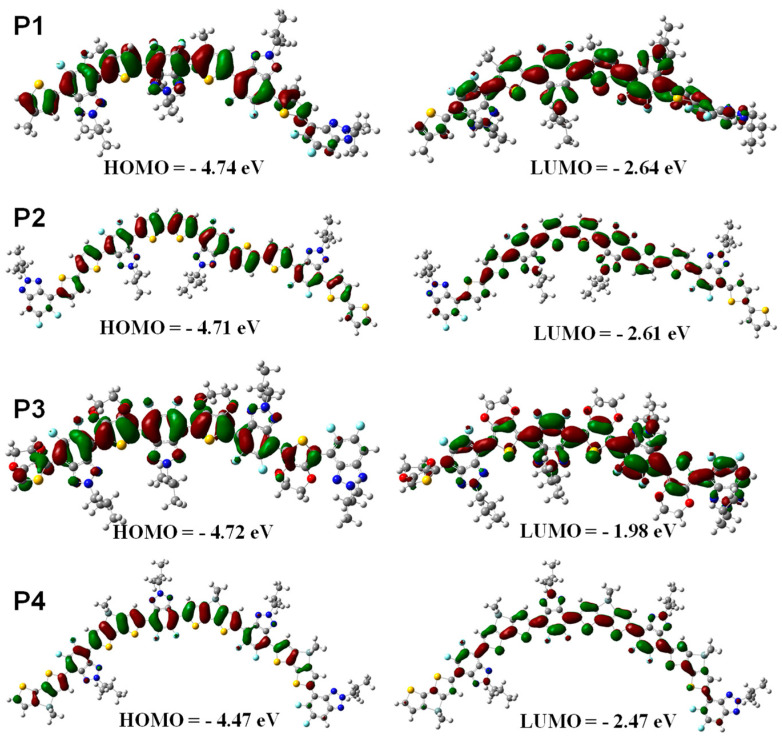
Optimized geometries and molecular orbital surfaces for the HOMO and LUMO values of **P1**–**P4** obtained using a DFT/B3LYP/6-31G* method.

Density functional theory (DFT) simulation was performed at the B3LYP/6-31G* level using Gaussian 09 programs to check the effect of the structure of thiophene donor units on the polymer backbone on the HOMO and LUMO energy levels of the resultant polymers. In this study, four repeated D-A units were used and all side chains were replaced with methyl groups to simplify the calculation. The HOMO and LUMO level values were obtained by MO topologies on the basis of the optimized geometry. As shown in Figure 2, the contour plots indicated the effective separation of HOMO and LUMO levels for all polymers **P1**–**P4**, which is beneficial to charge-carrier transport. Thus, these polymers were suitable for active materials of OLED devices. Additionally, it can be clearly seen from the molecular orbital diagrams that the HOMO energy levels of the four polymers all remain delocalized along the main chain of polymers, and the HOMO energy levels of **P1-P4** are −4.74, −4.71, −4.72, and −4.47 eV, respectively. While the electron density is successively distributed on the whole D-A units, the LUMO values of **P1**–**P4** are calculated to be −2.61, −2.64, −1.98, and −2.47 eV, respectively. It is important to note that the thiophene segments tend to be trans-coplanar with benzotriazole units in the optimized geometries of **P1** (Figure 2), where the sulfur atom of the thiophene unit is in the opposite position to the fluorine atom of the thiazole unit. This conformation is undoubtedly conducive to the formation of F⋯H non-covalent interactions as demonstrated by ^1^H NMR spectra shown in Figure 1A.

### 3.4. UV–vis Absorption Spectra

**Figure 3:**
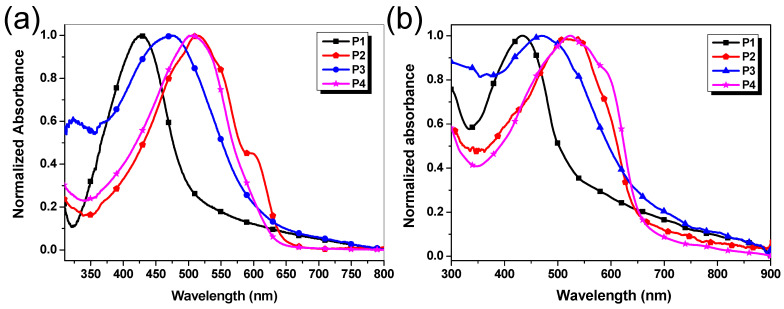
Normalized absorption spectra of **P1**–**P4** (**a**) in dilute chloroform solution (1 × 10^−5^ mol/L) and (**b**) in the thin film state (spin-cast from chloroform solution).

The UV–vis absorption spectra of **P1**–**P4** in dilute chloroform (CHCl_3_) solutions and in film states were measured on a Shimadzu UV-2550 spectrometer. The concentration of the polymer in CHCl_3_ solution was 10^−5^ mol/L based on repeated units. The film samples were prepared by casting the chloroform solutions (3 mg/mL) on the surface of quartz and subsequently annealing for 10 min at 100 °C. Their UV–vis absorption spectra of polymer solutions and thin films are presented in Figure 3, and the absorption maxima and the optical band gaps estimated from the absorption edge of the thin film are listed in Table 2.

As shown in Figure 3a and Table 2, the λ_max_ of **P1**–**P4** in CHCl_3_ solutions are 427, 515, 471, and 506 nm, respectively. Compared with **P1**, a remarkable redshift by 88 nm was observed for **P2** in CHCl_3_ solutions, possibly due to **P2** having longer conjugated chains and a more efficient conjugated effect caused by smaller steric hindrance between benzotriazole units and dithiophene units. In addition, the λ_max_ of all polymers in the thin film states exhibit negligible changes in comparison with that in solutions beside **P4**. The results should be attributed to only **P4** tending to form a more coplanar configuration between benzotriazole segments and silole ring in the solid-state due to the completely symmetrical and rigidly planar structure of silole units as demonstrated by DFT calculation (Figure 2). The optical band gaps (*E*_g_^opt^) of **P1**–**P4** were determined to be 2.16, 1.81, 1.85, and 1.88 eV, respectively, from the onsets of the spectra in the films. The results are close to the corresponding *E*_g_^opt^ values of the other benzotriazole-based D-π-A conjugated polymers reported in the literature, including ours [9,27].

### 3.5. Photoluminescence Property

**Figure 4:**
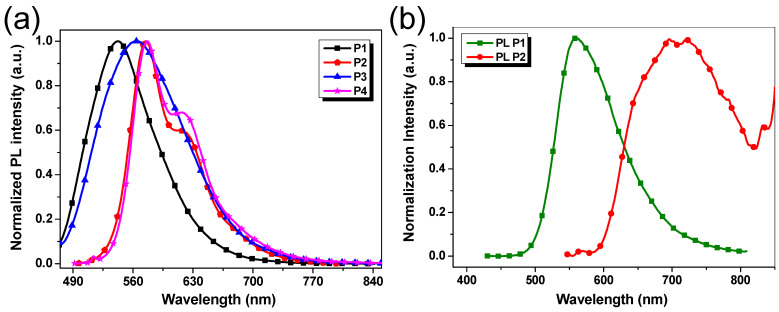
(**a**) Normalized PL spectra of **P1**–**P4** in dilute chloroform solutions (1 × 10^−5^ mol/L); (**b**) Normalized PL spectra in the thin film states of **P1** and **P2**.

Figure 4a shows the photoluminescence (PL) spectra in CHCl_3_ solutions. **P1**–**P4** exhibit broad emission peaks in the range of 480–770 nm upon excitation at 420 nm. In the case of **P2** and **P4**, the obvious shoulder bands at about 630 nm could be attributed to the intramolecular charge transfer (ICT) from thiophene units to benzotriazole units (Figure 4a) [38,39]. Additionally, it was noted that emission peaks of polymers vary with the different donor units, exhibiting a bathochromic displacement observed in the following order: **P1** < **P3** < **P2** ≈ **P4** (Figure 4a). Thus, the polymers **P2** and **P4** with the more extended conjugated backbones show the maximum emission peaks at the highest wavelengths between 550 and 650 nm (Figure 4a).

Different emission behavior of the polymers was noticed in solid-state. **P1** maintained a similar emission profile to that in solution but consisted of a larger spectral domain, extended to most of the visible light region from 500 to 700 nm. The solid-state **P2** displayed a very wide emission extended into the near-infrared domain, with an emission peak located at near 700 nm (Figure 4b). Compared with the emission peak of **P2** in solution, its significant red-shift in thin-film state was noted. This should be attributed to more effective conjugation of polymer chains due to the intramolecular rotation that was restricted in solid-state. For the films of **P3** and **P4**, only very weak emissions were observed (not provided), which agrees with the results of similar polymers reported in the literature [40].

### 3.6. Electrochemical Property

The electrochemical properties of all polymers **P1**–**P4** were assessed by standard cyclic voltammetry (CV) using a saturated calomel electrode as reference electrode and the energy level of ferrocene/ferrocenium (−4.80 eV) as the internal standard (Figures S10 and S11). The energy levels of **P1**–**P4**, calculated on the basis of CV curves, are presented in Table 2. The HOMO levels of **P1**–**P4** are −5.57, −5.69, −5.45, and −5.67 eV, respectively versus Fc/Fc^+^, and obviously lower than that of classical poly(3-hexylthiophene) (P3HT) materials (−5.10 eV) [41]. The results also indicate that the HOMO levels of benzotriazole-based conjugated polymers can be finely tuned by the introduction of different donor units. After magnification of the CV plots, the onsets of reduction potentials (*E*_onset_^red^) could be determined to be −0.73, −0.81, −0.82, and −0.83 V for **P1**–**P4**, respectively. Hence, the LUMO energy levels of polymers **P1**–**P4** can be obtained to be −3.60, −3.68, −3.59, and −3.58 eV, respectively, versus Fc/Fc^+^ according to the corresponding *E*_onset_^red^. Since the conjugated polymers containing fluorinated benzotriazole segments can display excellent oxidational stability due to the stabilized HOMO and LUMO levels by the strong inductive effect of fluorine atoms [11,42], such polymer materials should have a huge potential for application in various photoelectric devices.

### 3.7. Electroluminescence Characteristics

**Figure 5:**
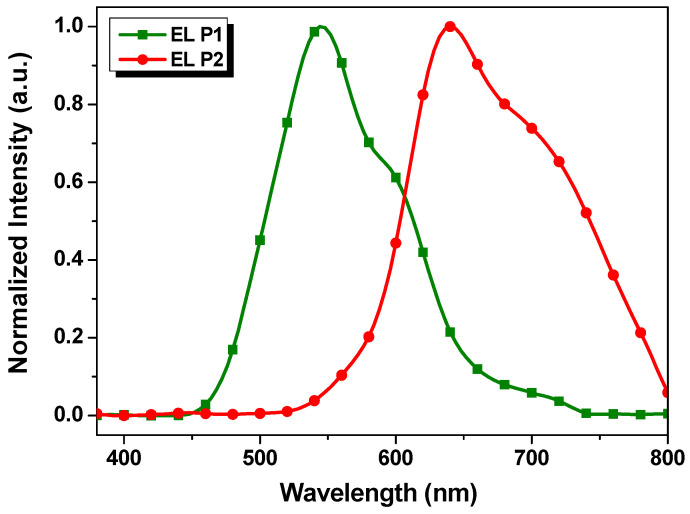
EL spectra **P1** and **P2**-based devices.

Considering that **P1** and **P2** have relatively high *M*_n_ and good fluorescent quantum efficiency in solutions (*η* = 0.3837 and 0.3805 for **P1** and **P2**, respectively, measured on a FLS1000 fluorescence spectrometer, Edinburgh, UK), the solution-processed OLED devices with the **P1** and **P2** as emissive layers were fabricated based on the following configuration: ITO/PEDOT:PSS/PTAA/emissive layer/TPBi/LiF/Al. Figure 5 shows the electroluminescent (EL) spectra of the devices with the **P1** and **P2** as the emissive layer, respectively. The emission maxima of **P1** and **P2**-based EL devices were 544 and 640 nm, respectively, which were slightly blue-shifted in comparison with the corresponding photoluminescence ones in the solid films as shown in Figure 4b. These results suggest that the color tuning on the EL device of benzotriazole-based polymers through the incorporation of various donor units on their backbone is feasible.

**Figure 6:**
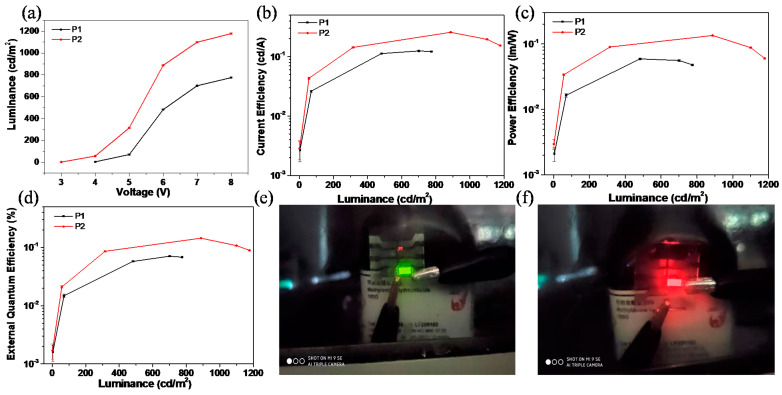
(**a**) The luminance–voltage (*L*–*V*) characteristics; (**b**) Current efficiency–luminance (*J*–*L*) curves; (**c**) Power efficiency *versus* luminance plots; (**d**) External quantum efficiency–luminance characteristics of **P1** and **P2**. (**e**) The photograph of **P1**-based device with green emission at 8 V; (**f**) The photograph of **P2**-based device with red emission at 8 V.

Figure 6 shows the typical EL performances of **P1** and **P2**-based devices and their EL characteristics that were also listed in Table 3. The rectification behavior of **P1** and **P2**-based devices can be observed from their *J*–*V* curves (Figure S12) originating from the intrinsic properties of the diodes. The turn-on voltages (*V*_on_) of **P1**- and **P2**-based devices were 4.0 and 3.0 V, respectively, as shown in Figure 6a. The **P2**-based device provided a higher luminance and current efficiency than that of **P1** at a certain bias, as shown in Figure 6a,b. As a result, the maximum power efficiency (*η*_p_ = 0.13 lm/W) and the maximum external quantum efficiency (EQE_max_ = 0.14%) for **P2**-based OLED devices, which is almost twice as much as that for **P1**-based devices (Figure 6c,d). The **P1**-based device exhibited a bright green emission with CIE coordinates of (0.40, 0.52) (Figure 6e), while a red emission with CIE coordinates of (0.66, 0.33) at 1200 cd/m^2^ was observed for **P2**-based devices (Figure 6f). This result indicates **P2** is a promising red emissive material for OLEDs.

## 4. Conclusions

Four benzotriazole-based π-conjugated polymers with donor-acceptor alternating structure were successfully prepared via Pd-catalyzed direct C-H cross-coupling polycondensation. The protocol offers a straightforward and effective assessment of such polymers without any pre-functionalized steps. This work extends the range of aromatic monomers for direct C-H cross-coupling polycondensation and achieves the effective copolymerization of electron-deficient benzotriazole with electron-rich thiophene derivatives in a structurally controllable mode. Although the as-synthesized polymer materials only displayed moderate OLED performances, the present method could have enormous potential for the synthesis of various semiconducting materials by tuning donor and acceptor units for different organic optoelectronic device applications.

## Figures and Tables

**Table 1:** Results of the polycondensation and the thermostability of **P1**–**P4**.

Entry	Polymer ^a^	*M*_n_^b^	*M*_w_^b^	*M*_w_/*M*_n_^b^	Yield/% ^c^	*T_d_* ^d^
1	**P1**	22,300	40,580	1.82	93	317
2	**P2**	28,100	34,280	1.22	82	385
3	**P3**	15,000	23,850	1.59	10	256
4	**P4**	3500	4690	1.34	20	392

^a^ Reaction conditions: benzotriazole monomer (1.0 equiv), thiophene monomer (1.0 equiv), Pd(OAc)_2_ (10 mol%), Ag_2_CO_3_ (3.0 equiv), K_2_CO_3_ (3.0 equiv), and PivOH (2.0 equiv) in DMAc/xylene (*v/v*, 10/1) for 48 h at 110 °C under N_2_; ^b^ Estimated by GPC measurements (eluent: THF; standard: polystyrene); ^c^ Isolated yield after purification. ^d^ The 5% weight-loss temperature under N_2_ atmosphere.

**Table 2:** Photophysical and electrochemical properties of **P1**–**P4**.

Entry	UV–vis			PL		CV		
	λ_max_^Sol^ (nm)	λ_max_^Film^ (nm)	$E_{g}^{opt}$ (eV) ^a^	λ_max_^Sol^ (nm)	λ_max_^Film^ (nm)	HOMO (eV) ^b^	LUMO (eV) ^b^	*E*_g_^cv^ (eV) ^b^
**P1**	427	428	2.16	542	560	−5.57	−3.60	1.97
**P2**	515, 596	510	1.81	575, 614	723	−5.69	−3.68	2.01
**P3**	471	468	1.85	564	-	−5.45	−3.59	1.86
**P4**	506	523	1.88	577, 616	-	−5.67	−3.58	2.09

^a^ Estimated from the absorption edge of the thin film. ^b^ Estimated from the cyclic voltammetry. *E*_HOMO_ = −(4.8 + *E*_onset_^ox^ − *E*_Fc/Fc+_^ox^) [eV]; *E*_LUMO_ = −(4.8 + *E*_onset_^red^ − *E*_Fc/Fc +_^red^) [eV]. *E*_g_^cv^ = *E*_HOMO_ − *E*_LUMO_.

**Table 3:** Luminescence characteristics of **P1** and **P2**-based OLED devices.

Polymer	λ_max_^EL^ (nm)	*V*_on_ (V)	Luminance (cd/m^2^)	*η*_c_ (cd/A)	*η*_p_ (lm/W)	EQE_max_ (%)	CIE (x, y)
**P1**	544	4.0	774	0.12	0.059	0.07	(0.40, 0.52)
**P2**	640	3.0	1178	0.25	0.13	0.14	(0.66, 0.33)

*V*_on_, voltage required for 1 cd/m^2^; *η*_c_, maximum current efficiency; *η*_p_, maximum power efficiency; EQE_max_, maximum external quantum efficiency; CIE, chromaticity coordinate.

## Data Availability

The data presented in this study are available on request from the corresponding author.

## Supplementary Materials

The supplementary materials are available online at https://www.mdpi.com/2073-4360/13/2/254/s1. Figures S1–S8: ^1^H NMR and ^19^F NMR Spectra of **P1**-**P4**; Figure S9: TGA curves of **P1**-**P4**; Figure S10: Cyclic voltammograms of **P1**-**P4**; Figure S11: Schematic energy-level diagrams of **P1**-**P4;** Figure S12: The current density-voltage curve of **P1** and **P2**-based devices.
