# Supplementary material for: Synthesis of π-Conjugated Polymers Containing Benzotriazole Units via Palladium-Catalyzed Direct C-H Cross-Coupling Polycondensation for OLEDs Applications
Source: Polymers (Basel). 2021 Jan 14;13(2):254. doi: 10.3390/polym13020254 (PMC7828650; doi:10.3390/polym13020254)
Supplement: Supplementary file 1 [file polymers-13-00254-s001.pdf]

Supporting Information

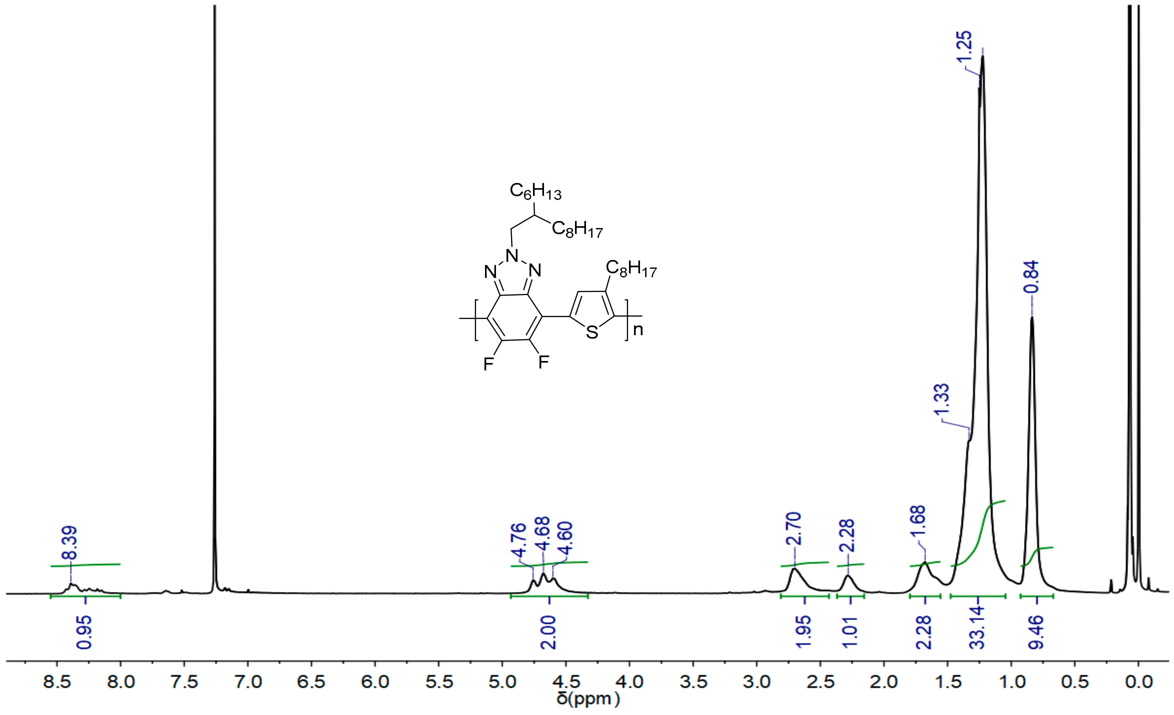

Figure S1. ^1H NMR spectrum of **P1** in $CDCl_3$.

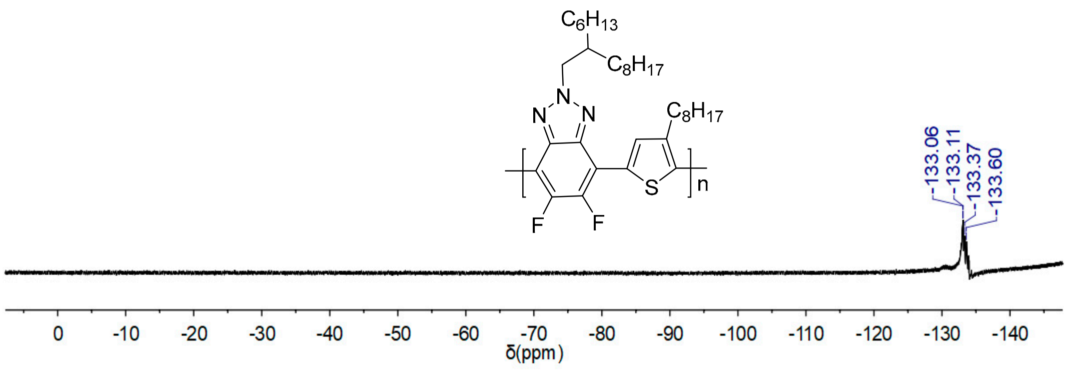

Figure S2. ^{19}F NMR spectrum of **P1** in $CDCl_3$.

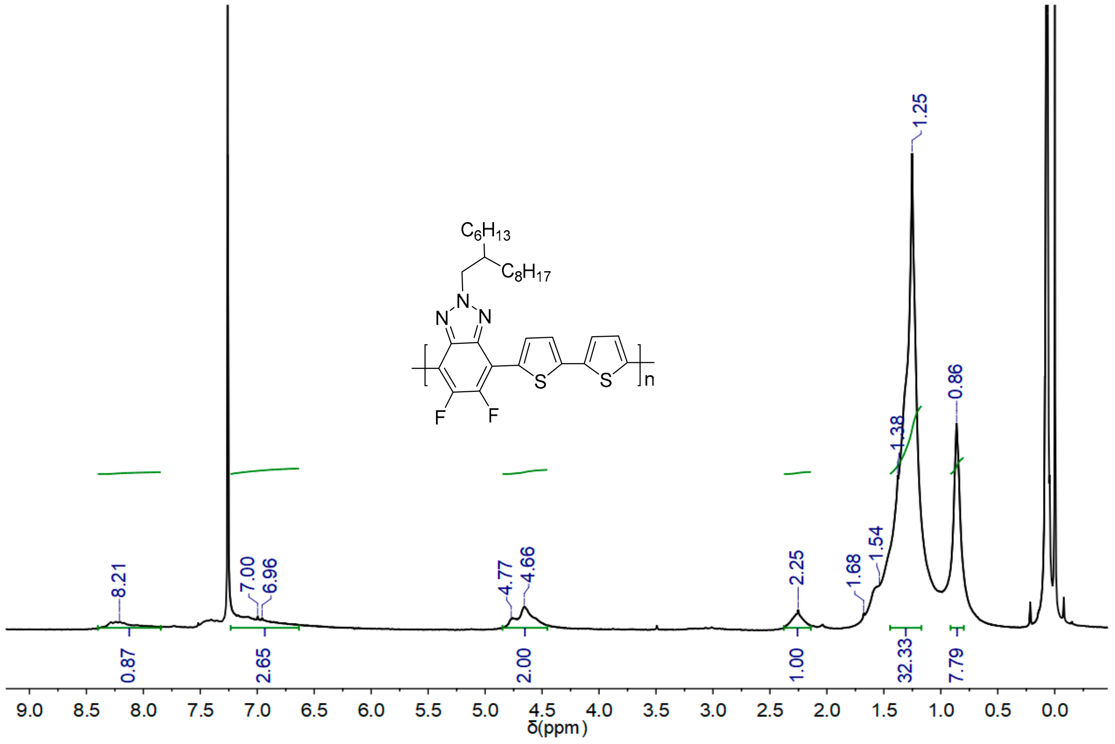

Figure S3. ^{1}H NMR spectrum of **P2** in $CDCl_3$.

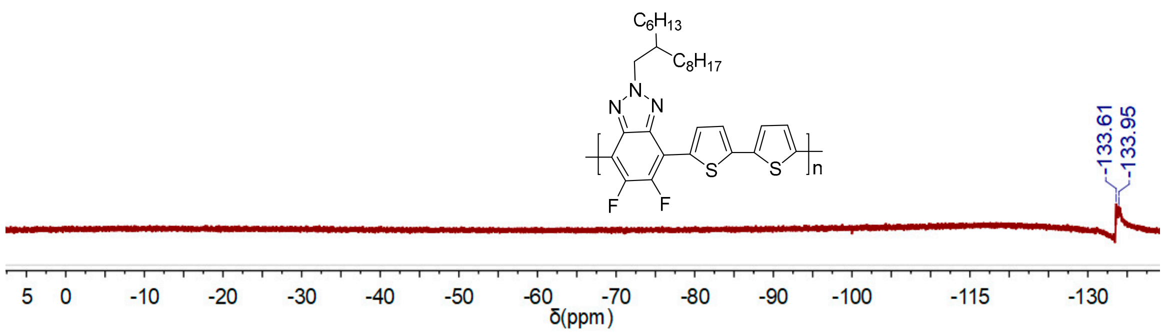

Figure S4. ^{19}F NMR spectrum of **P2** in $CDCl_3$.

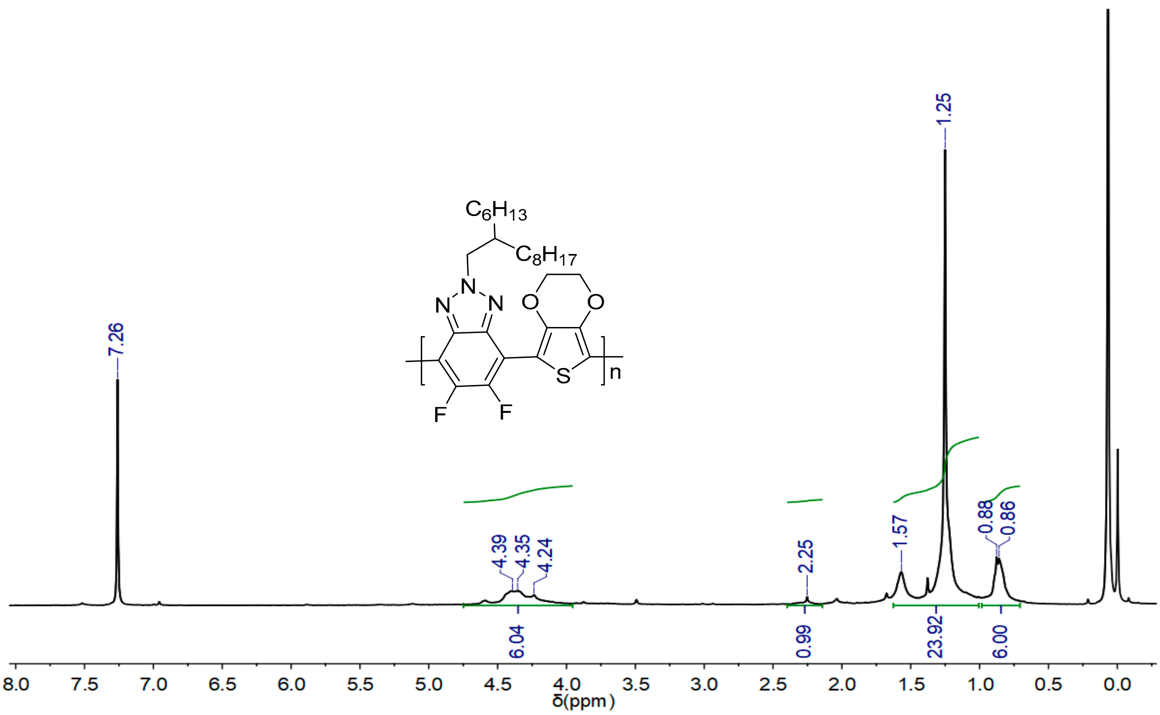

Figure S5. ^{1}H NMR spectrum of **P3** in $CDCl_3$.

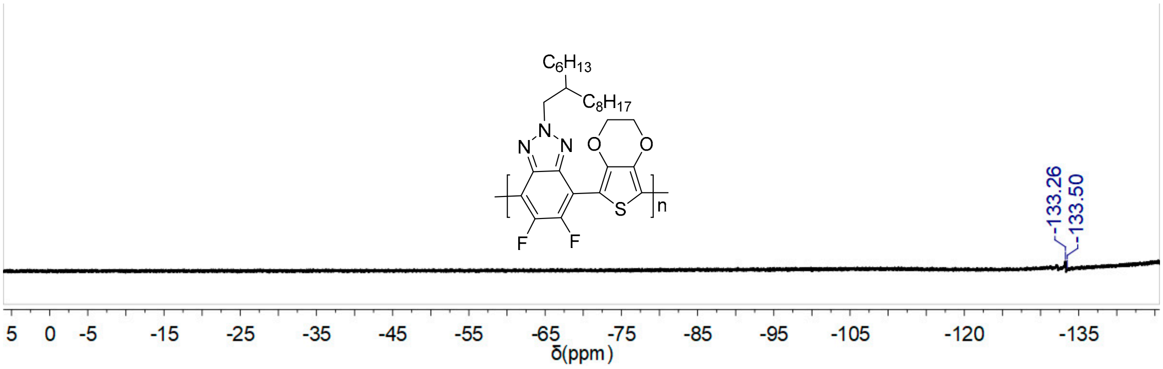

Figure S6. ^{19}F NMR spectrum of **P3** in $CDCl_3$.

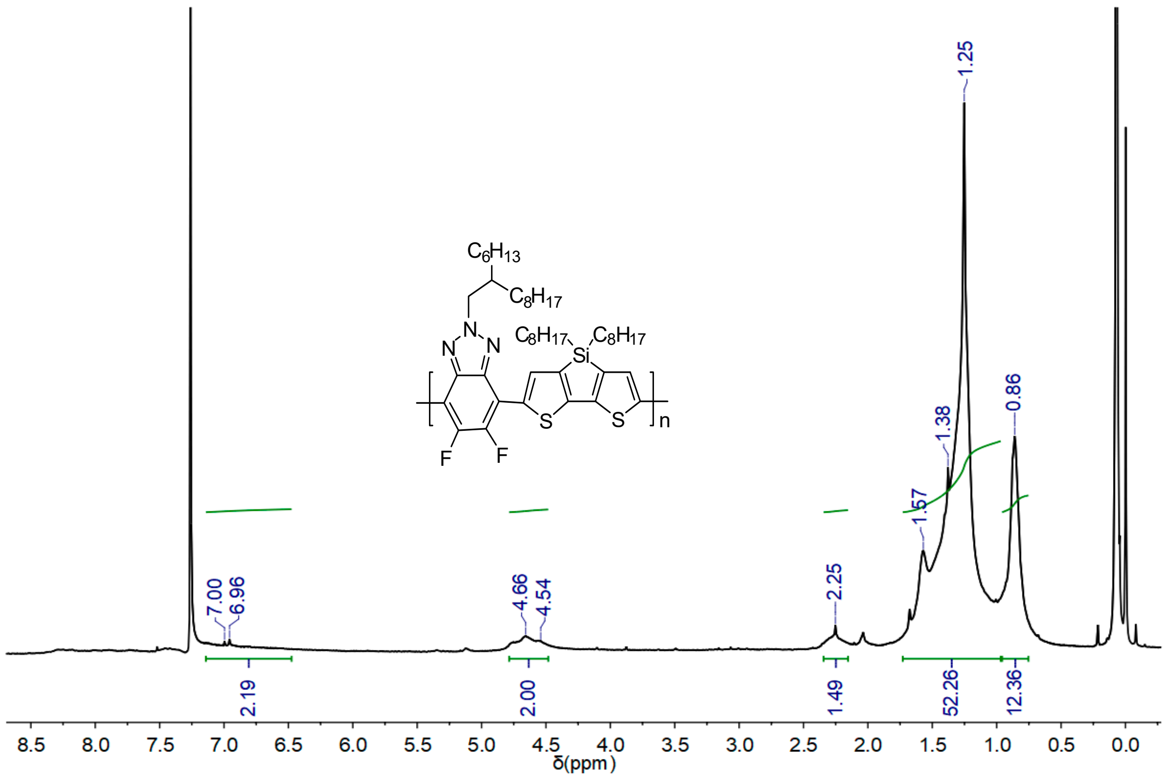

Figure S7. ^{1}H NMR spectrum of **P4** in $CDCl_3$.

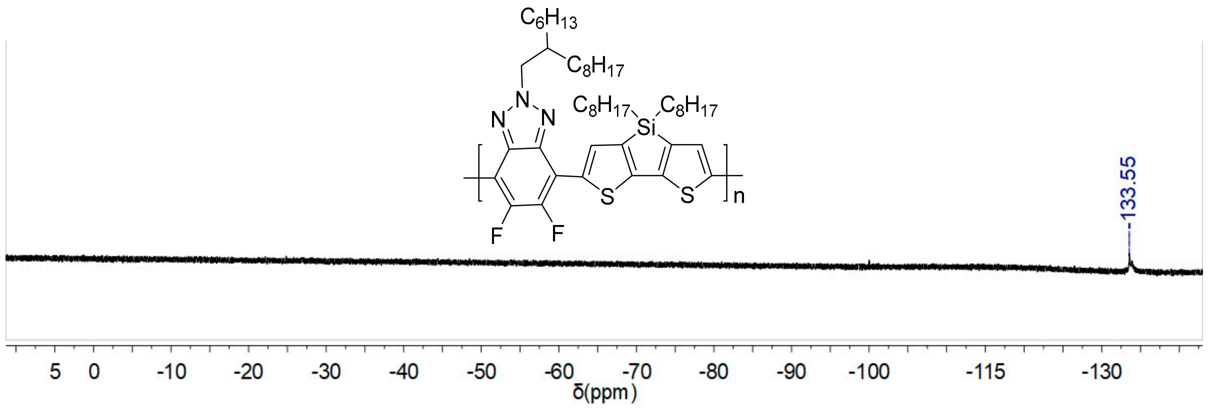

Figure S8. ^{19}F NMR spectrum of **P4** in $CDCl_3$.

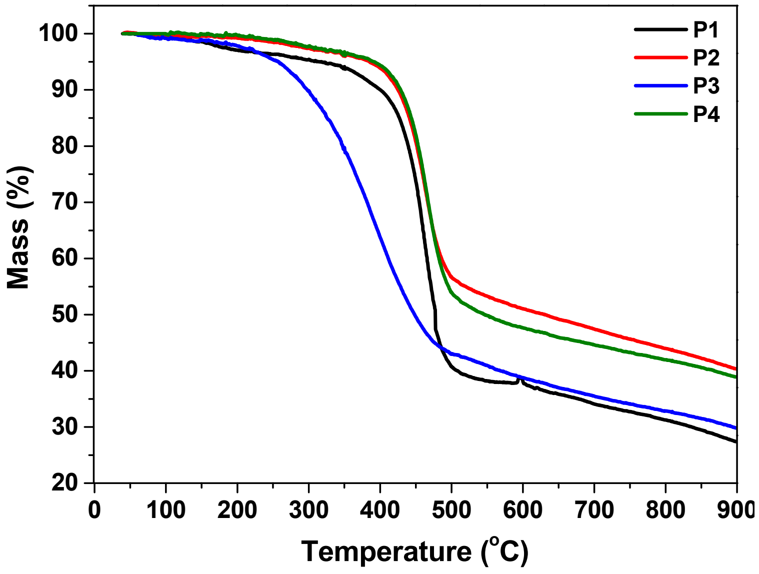

Figure S9. TGA curves of **P1-P4.** The heating rate is 10 °C·min^{-1} under pure nitrogen.

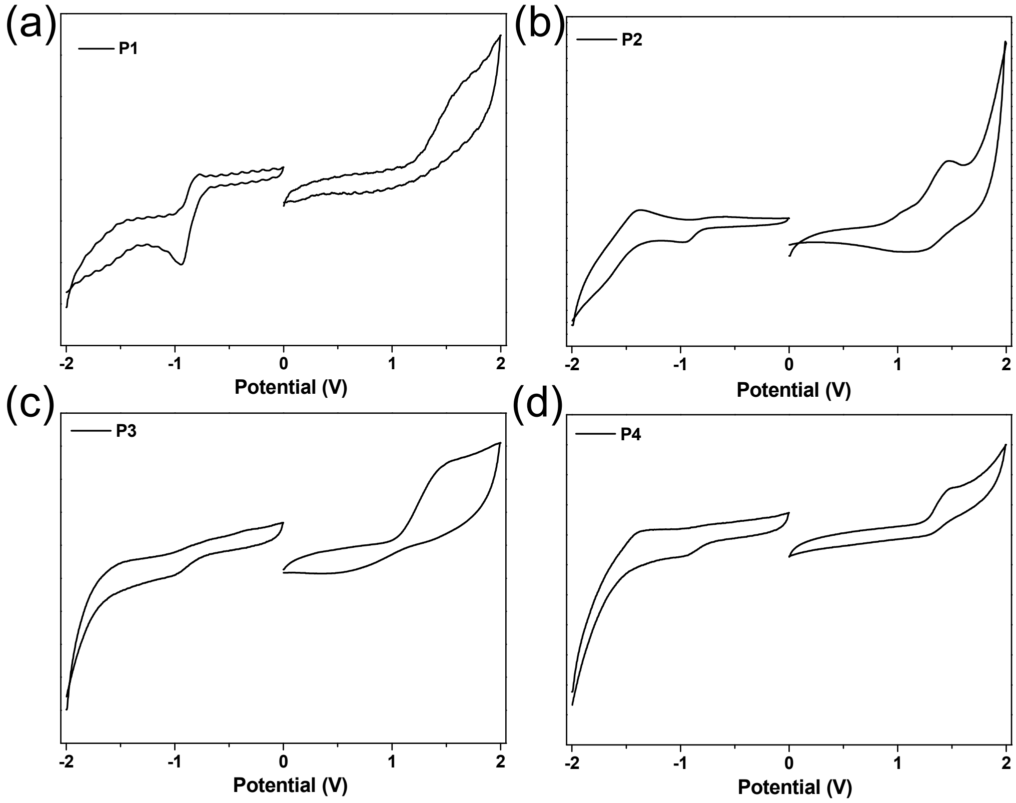

Figure S10. Cyclic voltammograms of **P1-P4** films on a glassy carbon electrode measured in 0.1 M of Bu_4NPF_6 acetonitrile solutions at a scan rate of 100 mV s^{-1}.

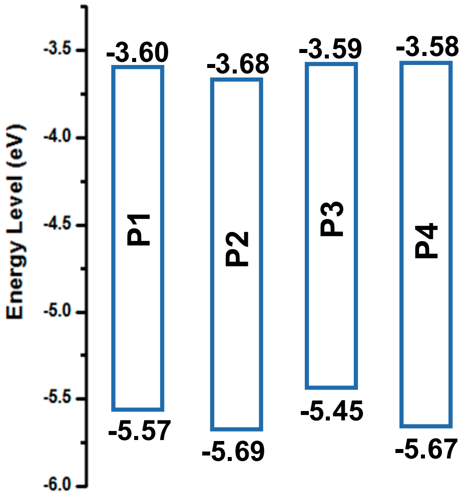

Figure S11. Schematic energy-level diagrams of **P1-P4.** Energy levels are estimated from the cyclic voltammetry (Figure S10). $E_{\mathrm{HOMO}} = -(4.8 + E_{\mathrm{onset}}{}^{\mathrm{ox}} - E_{\mathrm{Fc/Fc+}}{}^{\mathrm{ox}})$ eV; $E_{\mathrm{LUMO}} = -(4.8 + E_{\mathrm{onset}}{}^{\mathrm{red}} - E_{\mathrm{Fc/Fc+}}{}^{\mathrm{red}})$ eV. $E_g{}^{cv} = E_{\mathrm{HOMO}} - E_{\mathrm{LUMO}}$.

.

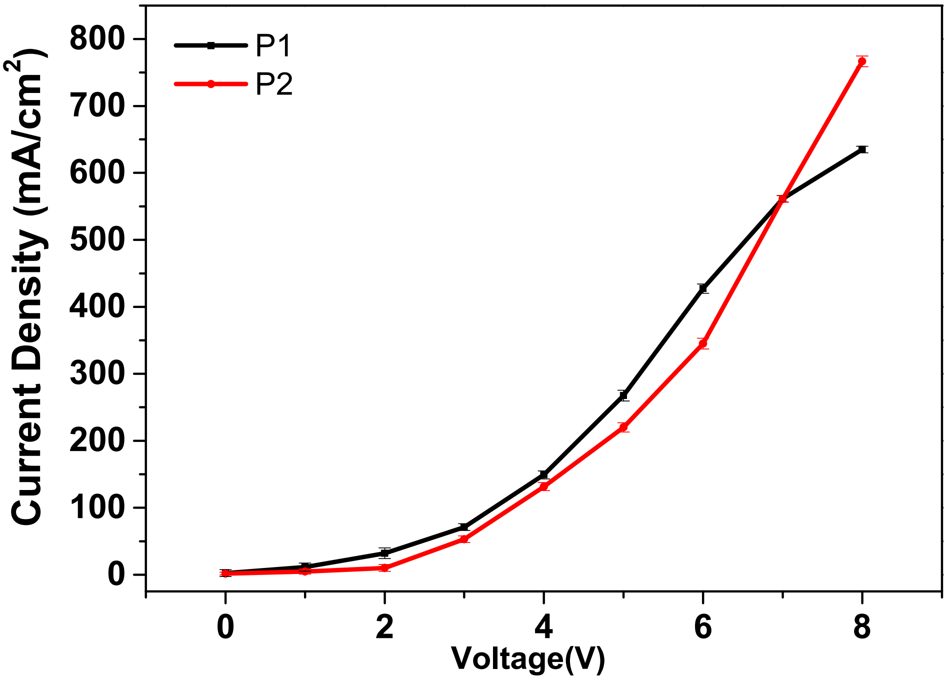

Figure S12. The current density-voltage curve of **P1** and **P2**-based devices.
